# Texture Recognition Based on Perception Data from a Bionic Tactile Sensor

**DOI:** 10.3390/s21155224

**Published:** 2021-08-02

**Authors:** Shiyao Huang, Hao Wu

**Affiliations:** State Key Laboratory of Digital Manufacturing Equipment and Technology, School of Mechanical Science and Engineering, Huazhong University of Science and Technology, Wuhan 430074, China; m201970670@hust.edu.cn

**Keywords:** tactile perception, vibration data, texture recognition, machine learning, convolutional neural network

## Abstract

Texture recognition is important for robots to discern the characteristics of the object surface and adjust grasping and manipulation strategies accordingly. It is still challenging to develop texture classification approaches that are accurate and do not require high computational costs. In this work, we adopt a bionic tactile sensor to collect vibration data while sliding against materials of interest. Under a fixed contact pressure and speed, a total of 1000 sets of vibration data from ten different materials were collected. With the tactile perception data, four types of texture recognition algorithms are proposed. Three machine learning algorithms, including support vector machine, random forest, and K-nearest neighbor, are established for texture recognition. The test accuracy of those three methods are 95%, 94%, 94%, respectively. In the detection process of machine learning algorithms, the asamoto and polyester are easy to be confused with each other. A convolutional neural network is established to further increase the test accuracy to 98.5%. The three machine learning models and convolutional neural network demonstrate high accuracy and excellent robustness.

## 1. Introduction

As an irreplaceable source of information, tactile perception is an important way of understanding the surrounding environment for humans [1]. Due to the fact that tactile perception is achieved through direct contact with objects, tactile data collection is only sensitive to the inherent physical characteristics of the object. This characteristic renders tactile perception highly reliable and less susceptible to external environmental interferences. Similar to humans, tactile perception in robots is essential to realize their intelligence. In the robotic system, tactile perception is achieved through the implementation of tactile sensors [2,3,4,5,6,7,8,9,10]. Similar as tactile perception of human, tactile perception of robots include many aspects such as discerning the shape, size, modulus, hardness, textures of objects, as well as contact forces and slippage during manipulation, etc. Among them, object texture recognition is important as the characteristics of object surface can significantly affect the grasping or manipulation strategies of robots.

In recent decades, the development of tactile sensors has greatly enhanced the ability of robotic tactile perception. Tactile sensors that measure temperature, humidity, contact force, or pressure have been demonstrated. Among those sensors, the contact force or pressure sensors are essential as the shape, size, texture, hardness of the object and slippage of grasping are usually judged based on the analysis of force data [11,12,13]. Contact force or pressure sensors based on various measurement mechanisms, such as piezoelectric sensors, piezoresistive sensors, triboelectric sensors, have been developed [14,15,16,17]. Tactile sensors that can only measure single-point contact pressure [18,19,20,21,22,23,24,25,26,27,28], or tactile sensor arrays with high spatial resolution [28,29,30,31,32,33,34,35,36,37,38], or those cover large area [39,40,41,42,43,44], have been developed. Commercially available tactile sensors, such as Biotac, which can simulate part of human perception and have high detection accuracy and stability, are also widely adopted for the recognition of object properties.

With the raw data collected from tactile sensors, it is necessary to propose effective algorithms for the classification of material properties such as surface texture. With the advances of machine learning [45,46,47,48] and deep learning [49,50,51,52], new solutions have been provided for interpreting tactile data. Fishel et al. [45] proposed a Bayesian exploration-based texture recognition method using three descriptive indicators of traction, roughness, and fineness. Through a number of well-chosen exploratory movements, the recognition success rate was 95.4%. However, this method required manual selection of the direction of exploration multiple times, which consumed a lot of time and labor. Yuan et al. [13] developed a surface material recognition algorithm based on tactile vibration and contact force. After the experimental data collection was completed, the method did not require any manual intervention, and only used the support vector machine model to achieve texture recognition, finally the overall texture recognition rate reached 80%. The neural network model can be used to extract data features directly, and the model can be trained to achieve classification and recognition. The deep learning algorithms have low manual participation and high accuracy, and they are widely used in many fields. Gandarias et al. [38] used two artificial intelligence methods to recognize objects through pressure images from high-resolution tactile sensors. The experimental results showed that the classification accuracy based on SURF was 80%, and the classification accuracy based on DCNN was 91.67%.

Although a variety of texture recognition methods have been proposed, it is still desirable to further improve the accuracy and reduce the computational costs of the algorithm. Therefore, in this study, we adopt the BioTac SP multi-modal tactile sensor to obtain the vibration signal generated when the sensor and the materials to be tested slide against each other. Based on the collected tactile signals, the material textures are identified through machine learning and deep learning methods, and the classification accuracy can reach as high as 98.5%. Compared with texture recognition methods reported previously, our approach features higher recognition accuracy with low computational time. The advantages and disadvantages of machine learning algorithms and deep learning algorithms in the classification of material texture are analyzed and evaluated. This work is organized as follows. Section 2 describes the collection of the data set and the algorithms used. Section 3 describes the results and discussion of the algorithms. The conclusions are given in Section 4.

## 2. Materials and Methods

**Figure 1:**
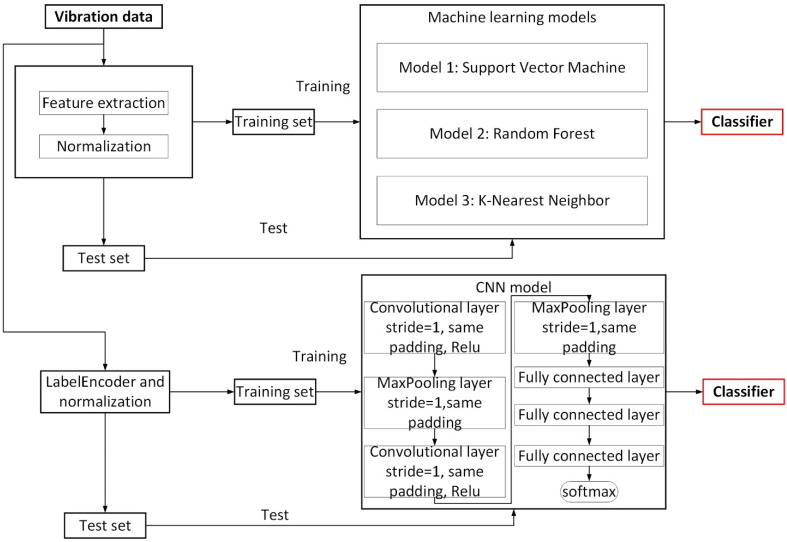
Flowchart of the proposed approach for texture recognition.

In this work, an experimental setup is built and the vibration data when the sensor and the material moved relative to each other under fixed experimental conditions are collected. Three machine learning algorithms and a deep learning algorithm are established to analyze and process the vibration data of different materials to achieve texture recognition of objects. In this paper, the term identification refers to detection and classification. The frequency domain features of the vibration data are extracted, and three machine learning models including Support Vector Machines, random forests, and K Nearest Neighbor are used to analyze the frequency domain features. A seven-layer convolutional neural network model is established, and the self-built data set is used to train the network model. A general flowchart of texture recognition is shown in Figure 1.

### 2.1. Experimental Device and Data Collection

**Figure 2:**
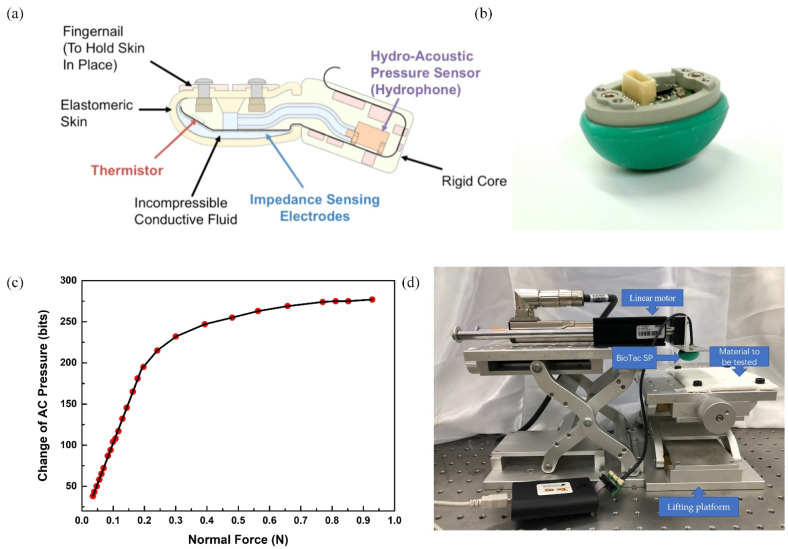
Experimental setup and measurement performances of the sensor: (**a**) schematic showing the structure of the original BioTac [55]; (**b**) photograph of BioTac SP sensor [55]; (**c**) the relationship between the change of AC pressure (bits) and applied normal force (N); (**d**) photograph of the experiment setup.

BioTac SP, a bionic sensor with high sensitivity and stability, was selected as the tactile sensor for vibration data collection in this study [53]. The original BioTac sensor structure is shown in Figure 2a. The biomimetic design consists of a rigid core surrounded by an elastic skin filled with liquid to provide compliance similar to the human fingertip. The photograph of BioTac SP is shown in Figure 2b. The design principle of BioTac SP is the same as the original BioTac but with slightly different structure design. The BioTac SP is capable of detecting the full range of cutaneous sensory information that human fingers can detect: forces, micro vibrations, and temperature [54]. The sampling rate of the sensor is 4.4 KHz. Row data collected from the BioTac SP include voltages on impedance sensing electrodes, absolute fluid pressure (DC Pressure), dynamic fluid pressure (AC Pressure), temperature (DC Temperature), and heat flow (AC Temperature). Among them, the AC pressure signal is related to the surface roughness and texture of the material to be tested, and can indicate the micro-vibration generated during the relative movement.

The AC pressure is measured with the BioTac SP sensor with a range of 0–4095 bits. In a small pressure range, the AC pressure is linearly related to the actual normal force (N), and the relationship between the change of the AC pressure and the normal force is shown in Figure 2c. Specifically, in order to characterize the relationship between the AC pressure displayed by the BioTac sensor and the actual applied force, a commercial force sensor (HP-50N by HANDPI) is used for calibration. The BioTac sensor is in contact with the force sensor, and they are subjected to the same magnitude of force at the contact position. In this way, the value indicated by the AC pressure in the BioTac is correlated to the commercial force sensor. By continuously changing the contact condition to vary the applied force, the correlation can be established as shown in Figure 2c. In order to ensure that the elastic skin of the sensor is not subject to severely wear during the data collection process, the experiment was carried out under the condition of a normal force of 0.15 N.

The photograph of the experiment setup for tactile data acquisition is shown in Figure 2d. The experiment setup is mainly composed of a LinMot linear motor, a lifting platform, a BioTac SP multi-modal tactile sensor and materials to be tested. The sensor is fixed at the end of the linear motor. We can change the height of the lifting platform to control the pressure between the sensor and the materials to be tested. The linear motor drives the sensor to move at a constant speed, and transmits the tactile sensor data collected during the movement to the host computer for processing.

**Figure 3:**
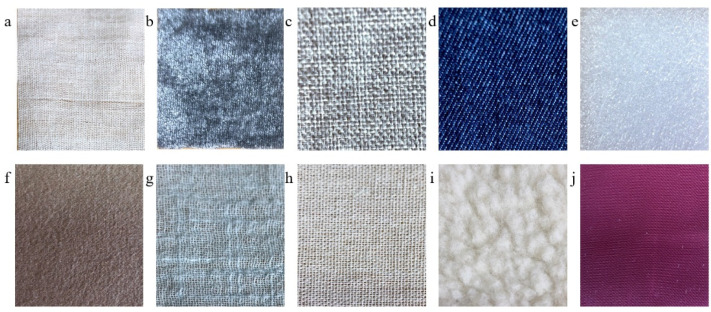
Photograph of ten materials tested: (**a**) polyester; (**b**) flannel; (**c**) asamoto; (**d**) denim fabric; (**e**) foam; (**f**) double-sided; (**g**) cotton linen; (**h**) flax; (**i**) lambswool; (**j**) satin.

**Figure 4:**
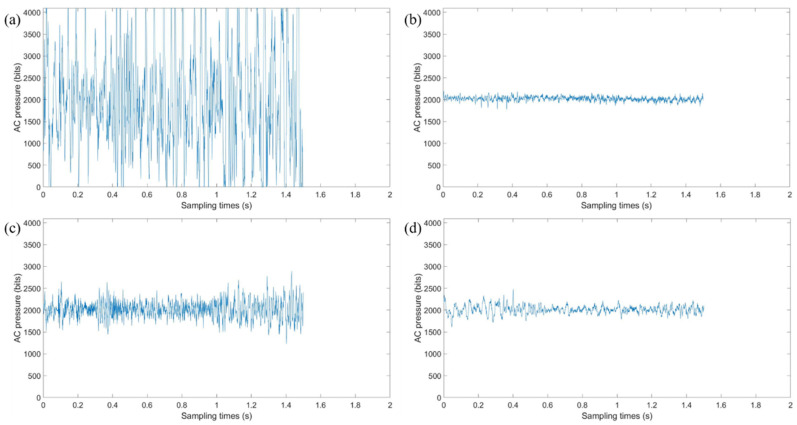
Vibration data of four materials: (**a**) polyester; (**b**) lambswool; (**c**) cotton linen; (**d**) foam.

We adjusted the height of the lifting platform to adjust the degree of contact between the BioTac SP sensor and the materials to be tested. The data were collected under a fixed pressure and speed. During the experiment, the tactile sensor maintained good contact with the sample which means the sensor is in close contact with the sample without separation, and the tactile sensor surface was not severely worn. The size of the materials to be tested was 4.5 cm × 5.5 cm. The linear motor was controlled to drive the sensor to move 3 cm at a speed of 0.01 m/s, and the vibration signal detected by the sensor was collected during this process. We adjusted the starting position of the sensor and repeated the movement. Vibration data are measured by multiple contacts in different areas and directions of each material. A total of 1000 sets of vibration data were collected from ten materials. The photographs of ten materials are shown in Figure 3, and the vibration signals of some materials are shown in Figure 4.

As shown in Figure 4, the vibration amplitude and frequency of the data collected from these materials are different, which is due to the different surface roughness and texture. Smooth surfaces are found to produce virtually no vibrations, while rougher surfaces produce vibrations of much greater amplitudes [22]. The lambswool has a smooth surface and the polyester has a rough surface, so that the vibration amplitude of polyester is much greater than the vibration amplitude of lambswool. The roughness of other materials is distributed between these two materials. Coarser textures are found to produce low-frequency vibrations, while finer textures produce higher-frequency vibrations [22]. The texture of asamoto is finer than the texture of lambswool, so that the vibration frequency of asamoto is higher than the vibration frequency of lambswool. The fineness of other materials is distributed between these two materials.

### 2.2. Feature Extraction of Vibration Data

The original vibration data have a large amount of data, and the sensor data are unstable in the acceleration and deceleration phases. The excessively large sample data have redundant information, and it is very easy to cause overfitting in training when it is completely input into the classification models. Therefore, the original data needs to be preprocessed and particular features need to be extracted to represent the characteristics of the data.

The setup of the hardware environment requires an Inter core i7-9750H processor at 2.60 GHz, with 16 GB RAM, 1T solid state drive, and NVIDIA GeForce RTX 2060 graphics card. The operating system is 64-bit Windows 10, and our programming software is MATLAB R2018b.

**Figure 5:**
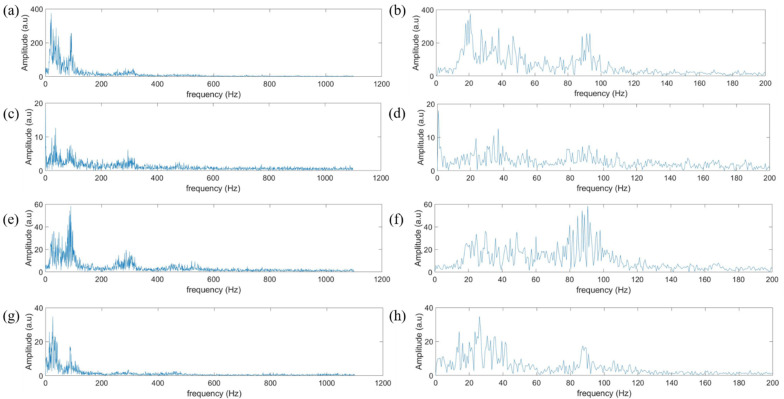
Frequency domain signals of polyester (**a**), lambswool (**c**), cotton linen (**e**), foam (**g**). The zoom in view of the signals in 0–200 Hz for polyester (**b**), lambswool (**d**), cotton linen (**f**), foam (**h**).

The fast Fourier transform (FFT) with rectangle window function was performed on the original vibration signals of different materials to obtain the frequency domain signal, as shown in Figure 5. It can be found that the frequency domain signal of the ten materials is distributed in 0–140 Hz, but the intensity distribution of different materials is rather different, so the feature can be extracted in the frequency domain for effective classification. The window length was set to 20 Hz, and the window was traversed from 0–140 Hz. Then we took the average intensity of each window as the feature, and each signal generated a seven-dimensional vector as the feature. Feature extraction of a set of data takes only 0.47 s.

### 2.3. Texture Recognition

The features of the vibration data of different materials are input to the models of support vector machine, random forest, K-nearest neighbor, and convolutional neural network.

The support vector machine is used to identify different textures based on vibration data. The vibration data collected in the experimental is non-linear and a few features are extracted. Therefore, the radial basis kernel function which is a nonlinear kernel is selected in this work. There are two hyperparameters in the model, namely the penalty coefficient of the support vector machine C and the radial basis kernel parameter gamma. These two hyperparameters are related to the generalization ability and time complexity. The penalty is optimized to adjust the tolerance of the model to errors, and the radial basis kernel parameter is adjusted to optimize the number of support vectors. The grid search algorithm is used to search and debug C and gamma. The accuracy of the algorithm reaches the highest when C is 30 and gamma is 0.1. A ten-fold cross-validation algorithm is used to test the accuracy of the model.

When using random forest to identify different materials, multiple decision trees need to be established, and the final result is voted by the recognition results of the decision trees. In the random forest model, it is necessary to determine the number of decision trees n_estimators and the maximum tree depth max_depth. When increasing the value of n_estimators and max_depth, the model may have better performance, but it will also increase the time complexity of the algorithm. Therefore, those two hyperparameters need to be adjusted appropriately. When the sample features are limited, the value of max_depth does not need to be limited. The grid search algorithm is used to search for these two parameters. When n_estimators is 100 and max_depth is not limited, the accuracy of the algorithm is the highest.

K-nearest neighbor is a supervised algorithm that can solve classification and regression problems. When using the K-nearest neighbor algorithm to identify different materials, it is necessary to determine the number of neighbor samples called K value, and the current sample label is voted by the neighbor sample label. The K value affects the accuracy and time complexity of the algorithm model, so adjusting K to an appropriate value is very important for the entire algorithm. The grid search algorithm is used to search for the value of the number of neighborhoods, and finally, when K is 5, the algorithm has the highest accuracy. A ten-fold cross-validation algorithm was used to test the accuracy of the model.

Convolutional neural network, a typical deep neural network, is widely used in the field of object detection and target recognition. A 7-layer convolutional neural network with 2 convolutional layers, 2 pooling layers, and 3 fully connected layers is also established to achieve texture recognition. The original data are reconstructed before being input into the convolutional layer, so that each sample in the original data is a 3300 × 1 vector. The structure of the convolutional neural network model is shown in Table 1. The first layer of the convolutional neural network is the convolutional layer, which has 8 convolution kernels of size 25, and the activation function is the Relu function. After the first convolutional layer, the output data size is 3300 × 8. The second layer is the pooling layer, which uses maximum pooling with a kernel of size 25, and the output data size is 220 × 8. The third layer is a convolutional layer, which has 16 convolution kernels of size 25, the activation function is the Relu function, and finally the output data size is 220 × 16. The fourth layer is the pooling layer, which uses maximum pooling with a kernel of size 15, and the output data size is 14 × 16. The output data are expanded and flattened, and a layer containing 224 neurons is obtained. After two fully connected layers, 10 neurons are output, which represent 10 different materials. Based on the output of 10 neurons, the type of material can be classified. The choice of hyperparameters in model training is very important to the final training results. The RMSprop optimization algorithm is used during training. The batch size is set to 16, the number of iterations, namely nb_epoch is set to 50, and the loss function of the model is Cross entropy loss function.

### 2.4. Performance Measures

The performance metrics used to compare the classifier performances are precision, recall, *F*_1_ score, and overall accuracy. These are computed from the confusion matrixes using Equations (1)–(4).

(1) $$Precision=\frac{TP}{TP+FP}$$

(2) $$Recall\left(TPR\right)=\frac{TP}{TP+FN}$$

(3) $$F_{1}\;Score=\frac{2TP}{2TP+FP+FN}$$

(4) $$Accuracy=\frac{TP+TN}{TP+FP+FN+TN}$$

*TP* refers to the positive samples that are predicted to be positive. *TN* refers to the negative samples that are predicted to be negative. *FP* refers to the negative samples that are predicted to be positive. *FN* refers to the positive samples that are predicted to be negative.

## 3. Results and Discussion

Support vector machine, random forest, and K-nearest neighbor algorithm models were built on the Jupter Notebook platform relying on the Sklearn Library, and they were used to classify ten materials.

In the process of training, it is necessary to divide the training set and the test set. Ten-fold cross-validation was used for the three machine learning algorithm models. The data set was divided into ten parts, and nine of them were used as training samples in turn, the rest was used as test sample. The average of the ten results was used as the estimation accuracy of the algorithm. The detection results of support vector machine, random forest, and K nearest neighbor algorithm are shown in Table 2.

**Figure 6:**
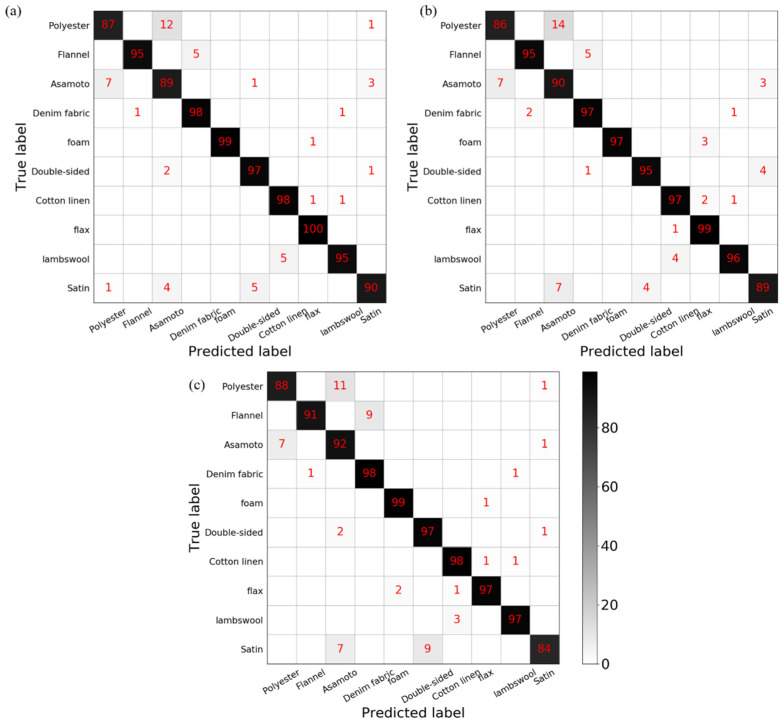
The confusion matrices of the three machine learning algorithms: (**a**) SVM; (**b**) RF; (**c**) KNN.

From the results of the three machine learning algorithms shown in Table 2, it can be seen that the three machine learning algorithms have high detection accuracy. For support vector machine, we can find that most of the test set data can be accurately classified, and only a few data are incorrectly identified. Among them, the most severely misidentified material is asamoto with an accuracy rate of 0.86. The recognition accuracy of materials except asamoto is above 0.9. According to the detection results of the random forest, we find that the recognition accuracy of asamoto is the lowest among all materials, and the recognition accuracy of other materials is above 0.9. The detection results of the K-nearest neighbor also show that the recognition accuracy of asamoto is the lowest among all materials, and the recognition accuracy of other materials is above 0.9. The confusion matrices of the three machine learning algorithms are shown in Figure 6. Among the three machine learning algorithms, most of the misclassified asamoto is judged to be polyester and most of the materials misjudged as asamoto are polyester.

**Figure 7:**
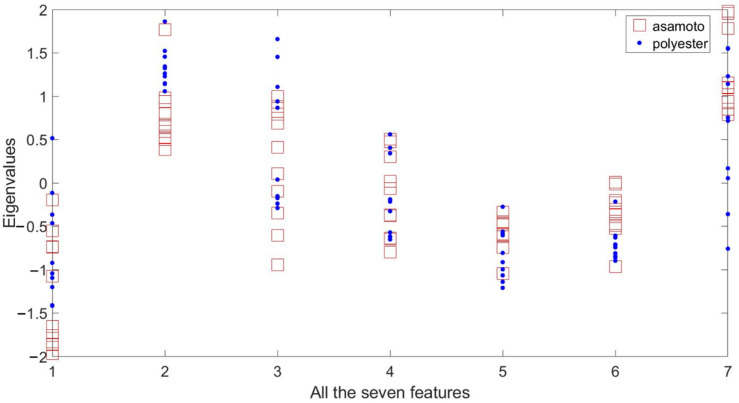
Features of asamoto and polyester.

The asamoto and polyester are easy to be confused with each other, and their features are shown in Figure 7. It can be found that all the seven features from the two materials overlap, thus they are easy to be misidentified. This is the reason the recognition accuracy of asamoto is not high when using the three machine learning algorithms.

We can see from the recognition results of the three machine learning algorithms that the recognition accuracy of asamoto is low, so other algorithms based on deep neural network need to be explored. The detection results of convolutional neural network proposed in this study are shown in Table 3.

**Figure 8:**
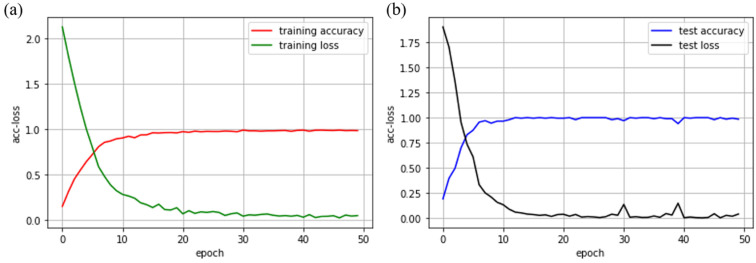
Training and test results of the convolutional neural network: (**a**) The change curve of training accuracy and training loss as the number of iterations increases; (**b**) the change curve of test accuracy and test loss as the number of iterations increases.

It can be seen in Table 3 that most materials can be accurately classified, and the recognition accuracy of all materials is above 0.9. The convolutional neural network solves the problem of low recognition accuracy of asamoto in machine learning algorithms. When using a convolutional neural network to realize object material recognition, the accuracy and training loss curves during training and testing are shown in Figure 8. The recognition accuracy and training time of the four algorithms are shown in Table 4. As the number of iterations increases, the training accuracy rate converges to 98.5%, and the test accuracy rate converges to 98.5%.

According to the training and test results, among the three machine learning algorithms, the support vector machine model has the highest recognition accuracy. The training time of the K-nearest neighbor algorithm is the shortest. This is because the result of the algorithm is only the bidding voting by the labels of the neighborhood samples, and no complicated mathematical operations are performed on the feature vectors. The training time of the random forest model is the longest. This is because the sample selection and node feature selection of each tree in the random forest are random, and there are two hyperparameters, namely the number of trees and the maximum tree depth. Each training requires exploring the number of trees and the depth of each tree to optimize the test accuracy. Five-fold cross-validation is adopted for all the machine learning algorithms. The variance of the accuracy obtained is shown in Table 4. It is observed that the variances are very low, indicating that the algorithms are very stable and robust. The test accuracy of the convolutional neural network is higher than that of the three machine learning algorithms, and the training accuracy of the convolutional neural network is 98.5%. Our method is compared with the methods proposed by other reports in recent years, and the results are shown in Table 5. It can be found that the algorithm we proposed has a higher recognition accuracy with low computational time.

Judging from the model training results, the convolutional neural network has very obvious advantages. In the process of establishing the model, the convolutional neural network can automatically extract features according to the settings of the model, without manually defining the types of features, rendering the establishment of the entire model simple and efficient. In the convolutional neural network, the concept of convolution kernel is also introduced, so that the convolutional layer has the characteristics of parameter sharing. The pooling layer reduces the number of neurons, and greatly reduces the number of parameters required to train the convolutional neural network. Therefore, the complexity and time required for training of the network is greatly reduced. The convolutional neural network achieves higher accuracy than machine learning methods.

## 4. Conclusions and Future Work

We have proposed object texture recognition methods based on tactile perception information from a bionic tactile sensor. Based on the vibration data collected by the sensor during sliding on material, the data preprocessing algorithm and feature extraction algorithm are proposed, four algorithm models of support vector machine, random forest, K nearest neighbor algorithm, and convolutional neural network are established, and training and test verification are completed. Experimental results show that compared with machine learning algorithms, convolutional neural networks have better recognition accuracy, which can reach 98.5%. Among the four algorithms, the K-nearest neighbor algorithm has a simple principle and does not involve complicated mathematical operations. The result is only obtained by voting on the neighborhood sample labels of the samples to be tested, so it has the most advantage in time efficiency. In the machine learning algorithm, our feature extraction is basically completed by addition, which reduces the time complexity of the entire algorithm. In the deep learning algorithm, our neural network has fewer layers, and the convolution kernel is not as complicated as 3D CNN, so the time complexity of the algorithm is relatively low. According to the results, our algorithm requires a shorter time. It has been shown that our approach features higher recognition accuracy with low computational time, when compared with texture recognition methods reported previously. In future work, the algorithm model can be further improved to improve the recognition accuracy of the algorithm. For deep learning algorithms, the upper limit of detection accuracy depends on the size of the data set, so the data set can be further expanded.

## Figures and Tables

**Table 1:** Operation, input, and output of each layer in CNN.

Layer Connection	Input Size	Operation	Kernel Size	Output Size
0–1	3300 × 1	Convolution	25 × 1 × 8	3300 × 8
1–2	3300 × 8	Maxpooling	15 × 1	220 × 8
2–3	220 × 8	Convolution	25 × 1 × 16	220 × 16
3–4	220 × 16	Maxpooling	15 × 1	14 × 16
4–5	14 × 16	Fully connected layer	224	224
5–6	224	Fully connected layer	128	128
6–7	128	Fully connected layer (softmax)	10	10

**Table 2:** Detection results of different materials using machine learning algorithms.

Algorithm/Material		TP	FP	FN	Precision	Recall	F1
SVM	Polyester	87	8	13	0.92	0.87	0.89
	Flannel	95	1	5	0.99	0.95	0.97
	Asamoto	89	18	11	0.86	0.89	0.87
	Denim fabric	98	5	2	0.95	0.98	0.97
	Foam	99	0	1	1.0	0.99	0.99
	Double-sided	97	6	3	0.94	0.97	0.96
	Cotton linen	98	5	2	0.95	0.98	0.97
	Flax	100	2	0	0.98	1.0	0.99
	Lambswool	95	2	5	0.98	0.95	0.96
	Satin	90	5	10	0.95	0.90	0.92
RF	Polyester	86	7	14	0.92	0.86	0.89
	Flannel	95	2	5	0.98	0.95	0.96
	Asamoto	90	21	10	0.81	0.90	0.85
	Denim fabric	97	6	3	0.94	0.97	0.96
	Foam	97	0	3	1.0	0.97	0.98
	Double-sided	95	4	5	0.96	0.95	0.95
	Cotton linen	97	5	3	0.95	0.97	0.96
	Flax	99	5	1	0.95	0.99	0.97
	Lambswool	96	2	4	0.98	0.96	0.97
	Satin	89	7	11	0.93	0.89	0.91
KNN	Polyester	88	7	12	0.93	0.88	0.90
	Flannel	91	1	9	0.99	0.91	0.95
	Asamoto	92	20	8	0.82	0.92	0.87
	Denim fabric	98	9	2	0.92	0.98	0.95
	Foam	99	2	1	0.98	0.99	0.99
	Double-sided	97	9	3	0.92	0.97	0.94
	Cotton linen	98	4	2	0.96	0.98	0.97
	Flax	97	2	3	0.98	0.97	0.97
	Lambswool	97	2	3	0.98	0.97	0.97
	Satin	84	3	16	0.97	0.84	0.90

**Table 3:** Detection results of convolutional neural network.

Algorithm/Material		TP	FP	FN	Precision	Recall	F1
CNN	Polyester	14	0	0	1.0	1.0	1.0
	Flannel	11	0	0	1.0	1.0	1.0
	Asamoto	25	0	0	1.0	1.0	1.0
	Denim fabric	24	0	0	1.0	1.0	1.0
	Foam	28	0	0	1.0	1.0	1.0
	Double-sided	16	1	0	0.94	1.0	0.97
	Cotton linen	16	0	2	1.0	0.89	0.94
	Flax	23	0	0	1.0	1.0	1.0
	Lambswool	21	2	0	0.91	1.0	0.95
	Satin	19	0	1	1.0	0.95	0.97

**Table 4:** Recognition accuracy of four algorithms.

Model	Training Accuracy (%)	Test Accuracy (%)	Time (s)
SVM	95.88 ± 1.48	95 ± 2.28	53.9
RF	95.59 ± 1.29	94 ± 2.24	273
KNN	96.75 ± 1.68	94 ± 2.24	3.76
CNN	98.5	98.5	25.9

**Table 5:** Comparisons of texture recognition algorithms.

Reference	Method	Sensor	Accuracy (%)	Run Time (to Train One Fold) (s)	Description
Strese et al. [56], 2014	Gaussian Mixture Model	Accelerometer	80.2	/	classification of 43 kinds of objects
Orii et al. [57], 2017	CNN	Pressure and 6-axis accelerometer	70.7	/	classification of 4 kinds of objects
Gandarias et al. [38], 2017	1. SURF	High resolution pressure sensor	80	0.01	classification of 8 kinds of objects
	2. DCNN		91.7	0.7	
Kerr et al. [58], 2018	SVM	BioTac	86.19	0.48	classification of 14 kinds of objects
Gandarias et al. [59], 2019	3D CNN whose input is 3D data	High resolution pressure sensor	96.3	/	classification of 9 kinds of deformable objects
Our method	CNN	BioTac SP	98.5	0.032	classification of 10 kinds of objects

## Data Availability

We did not report any data.
